# ABDOMINAL SCHWANNOMA UNVEILING LYMPHOMA: A RARE ASSOCIATION IN A COMPLEX CLINICAL PRESENTATION

**DOI:** 10.51894/001c.122912

**Published:** 2024-08-31

**Authors:** Peter Ng

**Affiliations:** 1 Family Medicine Ascension Genesys Hospital

## 44

### INTRODUCTION

Benign abdominal schwannomas are quite rare. While schwannomas can occur anywhere in the body, their occurrence in the abdominal region is relatively uncommon. Based on the current scientific literature available, there is limited information to suggest a link between abdominal schwannoma and T-cell lymphoma. Here, we report a unique case of an abdominal schwannoma that may have led to an unveiling lymphoma.

### CASE DESCRIPTION

We report a case of a 76-year-old female with a history of hypothyroidism, type 2 diabetes, and a previously diagnosed 4 cm abdominal schwannoma at the superior mesenteric artery level confirmed by biopsy. A remote history of uterine teratoma and no nonmalignant brain tumor was reported. The patient reported a 4-week history of abdominal discomfort with pressure and bloating. The patient sought evaluation for persistent nausea, accompanied by vomiting, palpitations, and fatigue. Additional complaints included recent diarrhea, nonproductive cough, shortness of breath, night sweats, and a 15-pound unintentional weight loss over three months. Despite no fever or hemoptysis, the complexity of symptoms raised concerns. Recent imaging with CT abdomen, chest and neck disclosed a 4.2 cm mass adjacent to the uncinate process with extensive lymphadenopathy in retroperitoneal, iliac, inguinal, mediastinal, bilateral hilar, and bilateral axillary regions. Severe upper cervical lymphadenopathy and enlarged palatine and lingual tonsils suggested lymphatic hypertrophy. Cancer-antigen 125 (CA-125) tumor marker was also elevated. A right axillary lymph node biopsy was performed and confirmed T-follicular helper (TSH) cell lymphoma, angioimmunoblastic type.

### CONCLUSION

This case underscores the rarity of a schwannoma possibly predisposing to lymphoma, revealing a complex interplay between neurogenic tumors and hematologic malignancies. Understanding such associations is crucial for timely diagnosis and multidisciplinary management in patients with intricate medical histories.

